# Molecular genetic characterization of p53 mutated oropharyngeal squamous cell carcinoma cells transformed with human papillomavirus E6 and E7 oncogenes

**DOI:** 10.3892/ijo.2013.1953

**Published:** 2013-05-24

**Authors:** JI-EUN OH, JEONG-OH KIM, JUNG-YOUNG SHIN, XIANG-HUA ZHANG, HYE-SUNG WON, SANG-HOON CHUN, CHAN-KWON JUNG, WON-SANG PARK, SUK-WOO NAM, JUNG-WOO EUN, JIN-HYOUNG KANG

**Affiliations:** 1 Laboratory of Medical Oncology, Research Institutes of Medical Science, The Catholic University of Korea, Seoul, Republic of Korea; 2 Division of Medical Oncology, Department of Internal Medicine, The Catholic University of Korea, Seoul, Republic of Korea; 3 Department of Hospital Pathology, Seoul St. Mary’s Hospital, The Catholic University of Korea, Seoul, Republic of Korea; 4 Division of Medical Oncology, Department of Internal Medicine, Uijeongbu St. Mary’s Hospital, The Catholic University of Korea, Seoul, Republic of Korea; 5 Department of Pathology, Microdissection Genomics Research Center, College of Medicine, The Catholic University of Korea, Seoul, Republic of Korea

**Keywords:** human papillomavirus, E6E7, head and neck squamous cell carcinoma, cDNA microarray, qRT-PCR array, JAK-STAT signal, STAT1, IGF-1R

## Abstract

Patients with HPV-positive oropharyngeal cancer show better tumor response to radiation or chemotherapy than patients with HPV-negative cancer. HPV oncoprotein E6 binds and degrades a typically wild-type p53 protein product. However, HPV16 infection and p53 mutation infrequently coexist in a subset of HNSCCs. The purpose of this study was to investigate the mechanisms through which tumor biology and molecular genetic mechanisms change when two HPV-negative, p53-mutated oropharyngeal cell lines (YD8, non-disruptive p53 mutation; YD10B, disruptive p53 mutation) derived from patients with a history of heavy smoking are transfected with HPV E6 and E7 oncogenes *in vitro*. Transfection with HPV E6 and E7 oncogenes in YD8, reduced the abundance of proteins encoded by tumor suppressor genes, such as p-p53 and p-Rb. Cell proliferative activity was increased in the cells transfected with E6E7 compared to cells transfected with vector alone (P=0.09), whereas the invasiveness of E6E7-transfected cells was significantly reduced (P=0.02). cDNA microarray of the transfected cells with E6E7 showed significant changes in mRNA expression in several signaling pathways, including focal adhesion, JAK-STAT signaling pathway, cell cycle and p53 signaling pathway. Regarding the qPCR array for the p53 signaling pathway, the mRNA expression of STAT1 was remarkably upregulated by 6.47-fold (P<0.05); in contrast, *IGF-1R* was significantly downregulated by 2.40-fold in the YD8-vector compared toYD8-E6E7 (P<0.01). Finally, data collected from these two array experiments enabled us to select two genes, *STAT1* and *IGF-1R*, for further study. In immunohistochemical study, nuclear STAT1 expression was slightly higher in HPV-positive compared to HPV-negative oropharyngeal tumors (P=0.18); however, cytoplasmic STAT1 was significantly lower in HPV-positive cases (P=0.03). IGF-1R expression levels were remarkably lower in HPV-positive compared to HPV-negative cases (P=0.01). Our data suggest that upregulated STAT1 and interferon signals by HPV16 E6 and E7 genes may play a major role in the relatively favorable prognosis for HPV-positive oropharyngeal squamous cell carcinoma cases with non-disruptive p53 mutations.

## Introduction

Head and neck squamous cell carcinoma (HNSCC) arises at the oral cavity, oropharynx, larynx or hypopharynx and is the sixth leading cancer by incidence worldwide (1). A subgroup of HNSCCs, particularly oropharyngeal carcinoma is caused by infection with high-risk types of human papillomavirus (HPV) (2,3). Compared with the HPV-negative tumors caused by heavy tobacco and alcohol use, the incidence of HPV-positive tumors has been recently reported to be strongly associated with sexual behavior, which is the predominant means of HPV transmission (4–6). The incidence of HPV-related oropharyngeal tumors has been increasing since the early 1990s in the United States and Western Europe, but the underlying reasons for this rapid increase are unclear (6,7). Human papillomavirus is a circular and double-stranded DNA virus. The viral genome encodes two regulatory proteins (E1 and E2), three oncoproteins (E5, E6 and E7) and two structural capsid proteins (L1 and L2) (8). The E6 oncoprotein forms complexes with a cellular E3 ubiquitin ligase (E6-associated protein; E6AP) and p53 protein, resulting in p53 degradation (9,10). The E7 oncoprotein binds to pRb family members and disrupts their ability to form complexes with E2F, increased expression of E2F-responsive genes, many of which are required for cell cycle progression (11,12). The E5 oncoprotein cooperates with E6 and E7 to promote proliferation of infected cells and is likely to facilitate malignant progression (13). However, the E5 coding sequence is frequently deleted from the episomal viral DNA during integration into the host genome (14).

Several recent studies have demonstrated that there are two distinct HNSCC etiologic risk groups: those who develop cancer in association with tobacco and alcohol and those who develop HNC as a result of HPV infection (2–4,15). The clinical outcomes after treatment with cisplatin and radiation therapy were significantly better in the patients with HPV-positive oropharyngeal carcinoma compared with those with HPV-negative carcinomas (16–18). Chemicals found in smoke, the major carcinogens responsible for HNSCC, are known to produce specific types of guanine nucleotide trans-version in crucial genes, such as the p53 tumor suppressor gene involved in the development of HNSCC (19). The p53 gene is mutated in up to half of HNSCCs (20,21), which are not infrequently found in HPV-positive oropharyngeal cancers (OPCs) (2,22).

A subset of the HPV-positive OPC patients with a history of extensive smoking have worse clinical outcomes than most HPV-positive OPC patients, resembling the clinical course in HPV-negative OPC patients. Considering that these patients overexpress *EGFR* and *Bcl-xL* and have a higher rate of TP53 mutation, it was proposed that HPV status alone is not an adequate prognostic marker for classifying patient groups (23). Based on these conflicting findings, the influence of tobacco in the development of HPV-associated HNSCC should be elucidated.

Given that the available research data was obtained from *in vitro* and *in vivo* HPV-positive tumor models unrelated to smoking history, it is necessary to apply the appropriate experimental models in order to understand a role of HPV in OPCs coexisting of HPV16 and p53 mutation. In this study, we investigated how tumor biology and molecular genetic mechanisms change when HPV-negative OPC cell lines bearing two different subtypes of TP53 mutations are transfected with HPV E6 and E7 oncogenes *in vitro*.

## Materials and methods

### Cell lines and culture conditions

Two HPV16-negative human squamous tongue cancer cell lines (YD8 and YD10B) and Caski cells (an HPV16-positive human squamous cervical cancer cell line) were obtained from the Korean Cell Line Bank (KCLB, Korean Cell Line Bank, Seoul, Korea). Both of the tongue cancer cell lines harbor p53 mutations, whereas the YD8 cell line has non-disruptive mutation that causes histidine to be substituted for by arginine at codon 273 in exon 8, the YD10B cell line has disruptive mutation that causes stop codon to be substitutes for tyrosine at codon 236 in exon 7 (27). The cell lines were cultured in RPMI-1640 (Welgene, Seoul, Korea) supplemented with 10% heat-inactivated fetal bovine serum (FBS; Gibco BRL, Grand Island, NY, USA), 1% penicillin/streptomycin (Gibco BRL), 10 mmol/l HEPES (Amresco Inc., Solon, OH, USA) at 37°C in a humidified incubator with 5% CO_2_.

### Transfection with HPV16 E6 and E7 oncogenes

We used both the HPV16 E6 and E7 oncogenes coding regions based on the sequences available from the HPV type 16 complete genome (GenBank: K02718.1). Both of the HPV16 E6 and HPV16 E7 oncogenes were amplified by polymer chain reaction (PCR). Primer sequences used to amplify a 776-bp PCR product were 5′-ATGCACCAAAAGAGAACTGC-3′ (sense) and 5′-TTCTGGTTTCTGAGAACAGAT-3′ (anti-sense). The PCR product was resolved in a 1.5% agarose gel and observed under ultraviolet light by staining with ethidium bromide after electrophoresis. We isolated a 776-bp DNA fragment containing HPV16 E6 and HPV16 E7 sequences and then cloned this in-frame within the CT-GFP Fusion TOPO^®^ (pcDNA3.1/CT-GFP-TOPO) of the mammalian expression vector pGFP (Invitrogen, Carlsbad, CA, USA), producing plasmid HPV16 E6E7. The resulting plasmids were purified by using a Plasmid Midi kit (Qiagen, Valencia, CA, USA) according to the manufacturer’s instructions and the presence of the correct inserts was confirmed by DNA sequencing (Cocmogenetech, Seoul, Korea).

For transfections, the YD8 and YD10B cells were plated in 6-well plates at a density of 1×10^3^ cells per well and allowed to grow overnight to 80–90% confluency. The following day, the cells were transfected with the mixture of 5 *μ*g plasmid DNA (the target sequence inserted plasmid HPV16-E6E7 and negative control plasmid) and 1.5 *μ*g of Xfect polymer nanoparticle (Clontech, Mountain View, CA, USA) in 2 ml of serum-free medium according to the manufacturer’s instructions. Four hours later, the medium was replaced by fresh growth medium. Cells were then incubated at 37°C in 5% CO_2_ in humidified chambers for 24 h. Transfectants were then selected using G418 antibiotic (Abm, BC, Canada), added dropwise to the culture medium to final concentrations ranging from 100 to 400 *μ*g/ml (YD8-E6E7, YD10B-E6E7). As negative controls, we used cells transfected with CT-GFP Fusion TOPO vector alone (YD8-V, YD10B-V).

### Western blotting

Cells were harvested with trypsin/EDTA, washed twice with PBS and lysed with RIPA cell lysis buffer (Gibco BRL) that contained a protease inhibitor cocktail (Amresco). Protein (30 *μ*g) from each cell type was used in Bio-Rad detergent-compatible protein assays (Bio-Rad Laboratories Inc., Hercules, CA, USA); proteins were resolved on 8–12% polyacrylamide gels using standard sodium dodecyl sulfate polyacrylamide gel electrophoresis (SDS-PAGE) and transferred onto polyvinylidene difluoride (PVDF) membranes (0.45 *μ*m; Millipore Corp., Billerica, MA, USA). Membranes were blocked with 5% skim milk (Becton-Dickinson, NJ, USA). Blots were then probed with the following antibodies; total Rb (Cell Signaling Technology Inc., Danvers, MA, USA), phospho-Rb (Ser^807/811^) (Cell Signaling Technology Inc.), E2F-1 (Cell Signaling Technology Inc.), p16 INK4A (Cell Signaling Technology Inc.), CDK4 (Cell Signaling Technology Inc.), phospho-p53(Ser^392^) (Epitomics Inc., Burlingame, CA, USA), cyclin D1 (Cell Signaling Technology), phospho-PTEN(Ser^380^) (Millipore Corp.), STAT-1 (Epitomics) and GAPDH (Abcam, Cambridge, MA, USA). Horseradish peroxidase (HRP)-conjugated secondary antibodies were purchased from Santa Cruz (Santa Cruz Biotechnology Inc., Santa Cruz, CA, USA). Blots were developed with an enhanced chemiluminescence reagent (Amersham Pharmacia Biotech Inc., Piscataway, NJ, USA) and detected using the LAS 3000 Image analyzer system (Fujifilm, Tokyo, Japan).

For western blot analysis, cells were harvested and analyzed for the expression of STAT-1 and IGF-1R. Total protein lysates were obtained and western blotting was performed as described previously. The antibodies recognized rabbit monoclonal STAT-1 (Epitomics) and rabbit polyclonal IGF-1R antibody (Epitomics). Protein expression was normalized against GAPDH expression (Abcam). Images were acquired with the LAS 3000 Image analyzer system (Fujifilm) and analyzed using the software provided by the manufacturer.

### Cell proliferation and invasion

Cell proliferation was measured using the 3-(4,5-dimethylthiazol-2-yl)-5-(3-carboxymethoxyphenyl)-2-(4-sulfophenyl)-2H-tetrazolium (MTS) assay. Cells were plated in a 96-well plate at 2×10^3^ cells per well. For 5 days, cells were incubated with 10 *μ*l of MTS/phenazine methosulfate (PMS) reagent for 4 h at 37°C in a 5% CO_2_ incubator. Following incubation in MTS, viable cells were counted every day by reading the absorbance at 490 nm using enzyme-linked immunosorbent assay (ELISA) reader (Spectra Max 250; Molecular Devices, Sunnyvale, CA, USA). Cell viability was calculated using Excel (Microsoft, Albuquerque, NM, USA) and expressed as the percentage of MTS absorption: % survival = (mean experimental absorbance/mean control absorbance) × 100. Data represent the mean ± SD.

The migratory potential of cells was evaluated using a 24-well format insert with 8-*μ*m pores (Becton-Dickinson, Franklin Lakes, NJ, USA). For the invasion assay, 1×10^5^ cells in serum-free medium were added to each upper insert pre-coated with matrigel matrix (BD, NJ, USA) and 750 *μ*l 10% FBS medium was added to the matched lower chamber. After 24-h incubation, cells that remained in the upper chamber were removed from the upper surface of the transwell membrane with a cotton swab and migrated to the bottom of the upper membrane surface were fixed in methanol, stained with Diff-Quik™, captured and counted. For migration assay, the procedures were similar, except that 1×10^5^ cells were added into the inserts without matrix gel pre-coated. Five random fields at ×200 magnification for each insert were counted. Inserts were conducted in triplicate in three separate experiments. The percentage of invasion was calculated as the mean number of cells invading through matrigel insert membrane/mean of cell migrating through control insert membrane x 100. Invasion was expressed as the invasion index, which was calculated as % invasion by HPV16 E6E7 transfected cells/% invasion by vector-alone cells.

### Cell cycle analysis

Cells were seeded at 5×10^5^ in 100-mm plastic dishes (Techno Plastic Products AG) and incubated for 72 h. The cells were trypsinized, washed twice with PBS and harvested by centrifugation. Briefly, cells were fixed with ice cold 70% ethanol for ≥1 h, centrifuged, washed twice in cold PBS, resuspended in 1 ml PBS and stained with propidium iodide (PI) solution (0.05 mg/ml PI, 10 mg/ml RNase A) for 20 min at 37°C in the dark. The fluorescence intensity was measured using a flow cytometer (FACSCalibur; Becton-Dickinson Biosciences, San Jose, CA, USA); at least 1×10^4^ cells were counted and DNA contents were analyzed using CellQuest software (Becton-Dickinson, Franklin Lakes, NJ, USA). All experiments were performed in triplicate. Data represent the mean ± SD. Statistically significant differences between the control and treatment groups were accepted at P<0.01.

### RNA isolation and cDNA microarray analysis

Total RNA was isolated from all the cells grown to 90% confluency using the TRIzol reagent (Invitrogen) according to the manufacturer’s instructions. Briefly, total RNA was extracted from cell lysate by phase separation with chloroform and RNA precipitation with isopropanol. After washing with 70% alcohol, the RNA was eluted in RNase-free water. Total RNA was quantified using a NanoDrop ND-1000 Spectrophotometer (NanoDrop Technologies Inc., Wilmington, DE, USA). A quality control test of total RNA was performed using the Experion™ system (Bio-Rad). Total RNA was cleaned up using Ambion columns (Illumina Total-Prep RNA Amplification kit, Ambion). Microarray analysis was performed using an Illumina HumanHT-12 v4 Sentrix Expression BeadChip (Illumina, San Diego, CA, USA). After hybridization of the biotinylated cRNA to the chips, the chips were scanned according to the standard protocol (Illumina). The arrays were scanned on the Illumina BeadArray reader, a confocal-type imaging system with 532 (Cy3) nm laser illumination. Data from each sample was extracted with Genome Studio software (Illumina) using default parameters.

### Quantitative real-time PCR (qRT-PCR) array

For the qRT-PCR array, we selected the Human p53 Signaling Pathway RT^2^ Profiler™ PCR Array (PAHS-027; Qiagen) for 84 genes relative cell proliferation, cell cycle, apoptosis. Total RNA was isolated from YD8-HPV and YD8-V cells grown to 90% confluency using the RNeasy Mini kit (Qiagen) according to the manufacturer’s instructions. Total RNA was quantified using a NanoDrop ND-1000 Spectrophotometer (NanoDrop Technologies Inc.). Reverse transcription was performed using the RT^2^ First Strand kit (Qiagen) as described by the manufacturer and carried out with RT^2^ Fast SYBR® Green qPCR Mastermix (Qiagen) using a Bio-Rad CFX96 system (Bio-Rad). The cycling conditions comprised 10-min enzyme activation at 95°C, followed by 40 cycles at 95°C for 15 sec, 55°C for 30 sec and 72°C for 30 sec. The complete data set obtained from the array analysis upload Excel Spreadsheet at http://pcrdataanalysis.sabiosciences.com/pcr/arrayanalysis.php (SABiosciences, Qiagen) and threshold cycle (Ct) value for each gene was used to calculate the fold-change in levels. Five housekeeping genes were included on the array to normalize the cDNA amounts: β-actin (*ACTB*), β-glucuronidase (*GUSB*), glyceraldehyde-3 phosphate dehydrogenase (*GAPDH*), heat shock protein 90 kDa α class B member 1 (*Hsp90ab1*) and hypoxanthine guanine phosphoribosyl transferase 1 (*HPRT1*). The formula used to calculate the relative gene expression level was (2^−ΔCt^). ΔCt = Ct (GOI) - avg. [Ct (HKG)], where GOI is the abundance of each gene and HKG are the housekeeping genes chosen from the ‘YD8-E6E7 Gene - YD8-V Gene’ worksheet. With the use of appropriate cut-off criteria, a 2-fold induction or repression of expression was considered to represent significantly up- or downregulated gene expression.

### Quantitative real-time PCR (qRT-PCR)

Quantitative real-time reverse transcriptase-PCR (qRT-PCR) was employed to validate genes that were differentially expressed by cDNA Microarray and qRT-PCR array. Synthesis of cDNA was performed with the Maxime RT PreMix kit (iNtRON Biotechnology, Korea) using 1 *μ*g of RNA in the reaction. FastStart Universal SYBR Green master mix (Roche, Mannheim, Germany) was added to the RT products and PCR was performed using a Bio-Rad CFX96 system (Bio-Rad). The primer pairs used for STAT-1 were 5′-CAAAGTCATGGCTGCTGAGA-3′ (forward) and 5′-AGGAAAACTGTCGCCAGAGA-3′ (reverse), whereas those for IGF-1R were 5-TGGAGTGCTGTATGCCTCTG-3′ (forward) and 5′-TGATGACCAGTGTTGGCTGG-3′ (reverse). The amplification program for all primer sets was 95°C for 5 min, followed by 40 cycles at 95°C for 30 sec, 60°C for 30 sec and 72°C for 1 min.

Assays were performed in accordance with the manufacturer’s instructions and the mRNA levels were normalized relative to levels of *GAPDH* transcripts. Relative expression levels of the mRNAs were calculated using the 2^−ΔΔCT^ values. Statistical analyses were performed using Microsoft^®^ Excel®. The average of triplicate real-time PCR measurements was used to calculate the mean induction ratio ± SD for each gene.

### Immunohistochemistry

Detection of HPV16-DNA was previously reported by *in situ* hybridization (ISH) methods (48). Samples were collected from 139 patients who underwent curative surgery for squamous cell carcinoma of the head and neck (HNSCC) in Seoul St. Mary’s Hospital between 1994 and 2009. The sites of HNSCC tumors included buccal mucosa (4 cases; 3%), tongue (66 cases; 47%), floor of the mouth (4 cases; 3%), soft palate (3 cases; 2%), tonsil (59 cases; 42%), oropharynx (2 cases; 1%) and uvula (1 case; 1%). To construct the tissue microarray block, tissue cylinders with a diameter of 2.0 mm, were taken from non-necrotic, morphologically representative areas of paraffin-embedded tumor tissues. Tissue cores from each specimen were assembled on a recipient paraffin block using a manual tissue arrayer (Quick-Ray Manual Tissue Microarrayer, Unitma Co. Ltd., Seoul, Korea). After construction, 4-*μ*m sections were cut and stained with hematoxylin-eosin staining on the initial slide for histological verification. Rabbit monoclonal anti-STAT1 (Epitomics) and rabbit polyclonal anti-IGF-1R (Epitomics) were used for immunohistochemical staining. Paraffin sections (4 *μ*m) from samples were deparaffinized in 100% xylene and re-hydrated in an ethanol series of decreasing concentrations of aqueous ethanol using standard protocols. Antigen was performed using the heat induced epitope retrieval method (HIER) in 0.01 M citrate buffer (pH 6.0). Endogenous peroxidase activity was blocked by immersion in 3% hydrogen peroxide in methanol for 10 min, followed by overnight incubation with rabbit monoclonal anti-STAT1 (1:200) and rabbit polyclonal anti-IGF-1R (1:100) at 4°C. After washing, the sections were incubated with polymer-conjugated horseradish peroxidase (HRP) for 10 min at room temperature. The peroxidase reaction was developed using 3,3-diaminobenzidine chromogen solution in diaminobenzidine (DAB) buffer substrate using polink-2 plus DAB detection kit (Two-step polymer-HRP detection system, biotin-free) (D43-15; Life Science Division, Mukiteo, WA, USA). Following incubation, the sections were visualized with DAB and counterstained with hematoxylin, mounted in neutral gum and analyzed using a bright field microscope.

The results were interpreted by a pathologist who was blinded to the specific diagnosis and prognosis for each case. The percentage of positive tumor cells was scored as follows: 0, no tumor cells stained; 1, 1–5% of cells stained; 2, 5–20% of cells stained; 3, 21–50% of cells stained; 4, 51–75% of cells stained; and 5, >75% of cells stained. The intensity of staining was scored as follows: 0, no staining; 1, low staining; 2, moderate staining; and 3, high staining. The immunoreactive score was calculated by multiplying the percentage of positive cells (scored 0–5) by staining intensity (scored 0–3). Tumors with an immunoreactive score were considered positive for STAT and IGF-1R expression. The total score was calculated by summing the percentage of positive cells and staining intensity values. For statistical analysis, a final staining was scored as follows: 0, negative; 1–4, low expression; 5 and 6, 8 and 9, intermediate expression; 10 and 12 and 15, high expression.

### Statistical analysis

Data were evaluated for statistical significance by analysis using Student’s t-test. A statistically significant difference was considered to be significant at P<0.05 or P<0.01. All experiments were performed independently at least three times and the data presented are from a representative experiment. The results are presented as mean ± SD.

## Results

### Expression of the E6E7 gene in two cancer cell lines transfected with HPV16 E6 and E7 oncogenes

**Figure 1:**
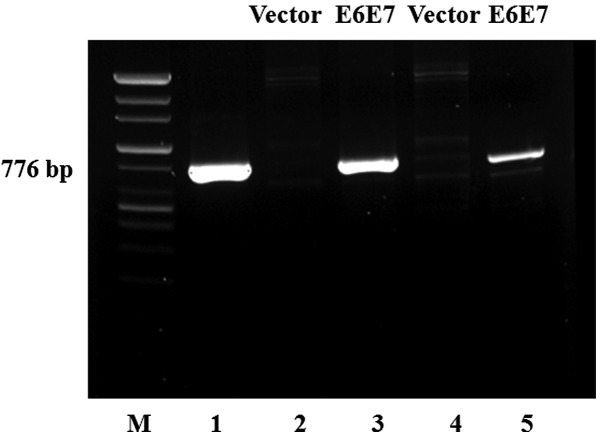
Confirmation that HPV16-negative cell lines expressed HPV16 E6 and E7 oncogenes only after transfection. We confirmed the E6E7 DNA amplifications both YD8 and YD10B cells expressed E6 and E7 oncogenes (YD8-, YD10B-E6E7; lanes 3,5), but did not express E6 and E7 oncogenes (YD8-, YD10B-vector; lanes 2 and 4). The Caski cell line (lane 1) provided a positive control. The HPV16 E6E7 PCR products were electrophoresed in a 1.5% agarose gel and visualized under ultraviolet light by ethidium bromide staining. M, molecular marker (100 bp ladder).

We confirmed the E6E7 DNA amplifications of the stable YD8 and YD10B cell lines transfected with HPV-16 E6 and E7 oncogenes. We performed PCR analysis using one primer pair specific to the HPV16 E6 and E7 oncogenes. Both YD8- and YD10B-E6E7 cells expressed the E6E7 gene. Neither YD8- nor YD10B-V expressed the E6E7 gene. We used Caski cell line as positive control (Fig. 1).

### Expression of p53- and Rb-related proteins

**Figure 2:**
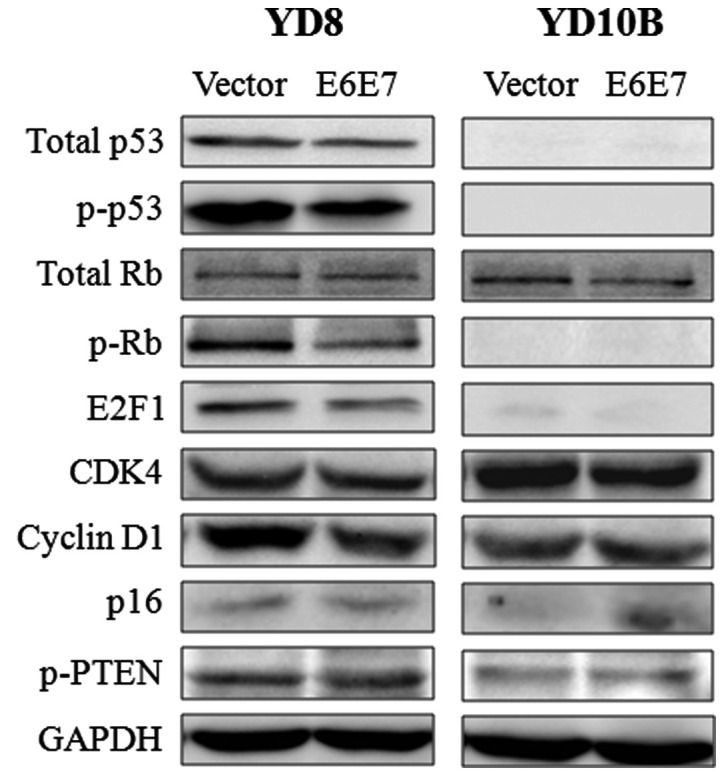
The expression of p53- and Rb-related proteins. The levels of expression of p53- and Rb-related proteins in YD8- and YD10B-E6E7 cells were analyzed using western blotting. The same blots were reacted with GAPDH antibody as loading control. Each experiment was performed in triplicate.

We next examined the biochemical responses of genes related to the p53 and Rb pathways in two cancer cell lines transfected with HPV16 E6 and E7 oncogenes. Although p53 protein was abundant in YD8 cells, levels were lower in YD8-E6E7 cells than YD8-V cells (40.6%). However, no expression of total p53 and p53 was observed in YD10B cells. Additionally, the levels of expression of pRb and E2F-1 were substantially lower in YD8-E6E7 cells than in YD8-V cells (38 and 33.7%) and were barely evident in either YD10B-E6E7 or YD10B-V cells. The level of expression of cyclin D1 was lower in YD8-E6E7 than in YD8-V cells (25.6%), but there was no difference in expression of cyclin D1 between YD10B-E6E7 and YD10B-V. The protein level of p-PTEN, total Rb and CDK4 were not considerably different in HPV16 E6E7 transfected cells and vector alone cells (Fig. 2).

### Differences in cell viability and invasive capacities of the two classes of transfected cells

To test our hypothesis that HPV infection alters the proliferative potential of oropharyngeal cancer cells, we compared cell proliferation activity in HPV16 E6E7 transfected cells and cells transfected with vector alone using the 3-(4,5-dimethylthiazol-2-yl)-5-(3-carboxymethoxyphenyl)-2-(4-sulfophenyl)-2H-tetrazolium (MTS) assay, which was carried out on days 0, 1, 2, 3, 4 and 5. The rate of proliferation of YD8-E6E7 cells was higher than that of YD8-V cells (43.9%, P=0.09) and YD10B-E6E7 cell proliferated at a rate higher than YD10B-V (38.1%, P=0.26).

**Figure 3:**
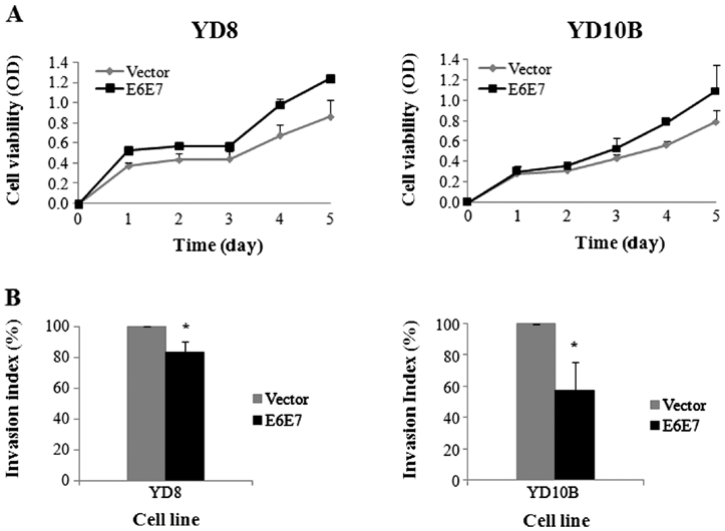
The differences of cell viability and invasion activity in two transfected cell lines. Cell viability and cell invasion were significantly different between HPV16 E6E7 transfected cells (YD8, YD10B-E6E7) and vector-alone cells (YD8, YD10B-V). Cells transfected with HPV16 E6E7 were more viable than cells transfected with vector alone (A) and cells transfected with HPV16 E6E7 had reduced invasion activity compared cells transfected with vector alone (B). Cell viability rate was calculated as the percentage of MTS absorption as follows: % survival = (mean experimental absorbance/mean control absorbance) x 100. Invasion activity was presented as the invasion index. The mean and standard deviation of the invasion index were then calculated. Each experiment was performed in triplicates. Data represent the mean ± SD. Statistically significant differences between the control and treatment groups are presented as ^*^P<0.05.

Proliferation of cells transfected with HPV16 E6E7 was higher than in cells transfected with vector alone. Especially, in YD8-E6E7 cells, cell proliferation increased rapidly from day 1 and then, gradually increased from day 2 to day 3 in YD10B cells (Fig. 3A).

Cells were seeded in the upper parts of the transwells. Invasion activity was expressed as an invasion index, which was calculated as the percentage of initial cell numbers attached to the bottom of a matrigel-coated membrane after 24 h. As shown in Fig. 3, we observed that invasion activity was significantly reduced in transfected cells with E6E7 compared with cells transfected with vector alone. The invasion activity of YD8-E6E7 cells was lower than that of YD8-V cells (17.1%, P=0.02) and invasion activity of YD10B-E6E7 was lower than that of YD10B-V cells (42.7%, P=0.05) (Fig. 3B).

### Changes in cell cycle distributions

Flow cytometry was used to show that the cell cycle distribution of cells transfected with HPV16 E6E7 was significantly different from that of cells transfected with vector alone. Compared with YD8-V values, the proportion of YD8-E6E7 cells in the G0/G1 phase was lower (66.1±31.5 vs. 71.9±1.2%; P=0.006) and the proportion in the G2/M was higher (18.3±1.2 vs. 13.9±1.4%, P=0.003). In contrast, the fraction of YD10B-E6E7 cells in the G0/G1 phase increased (79.4±2.8 vs. 74.5±2.8%, P=0.098) and the fraction in the G2/M phase decreased (12.3±0.7 vs. 16.4±0.4%, P=0.003) relative to YD10B-V values (Table I).

### Gene expression profiles of HPV E6E7 transfected cells

**Figure 4:**
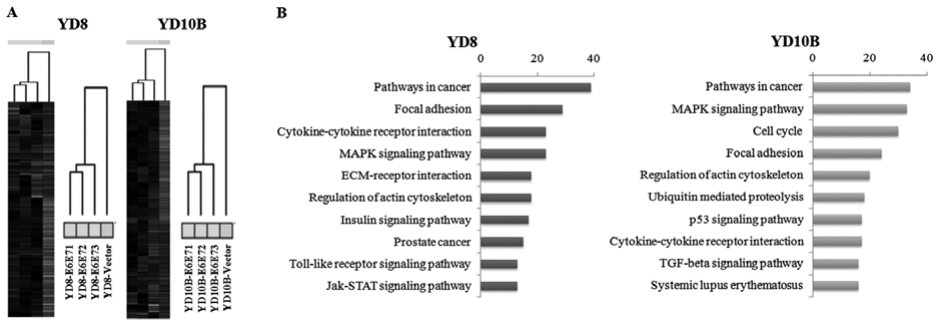
The gene expression profile in HPV E6E7 transfected cells. Two-dimensional hierarchical clustering analysis of expression profiling in cells transfected with HPV16 E6E7 compares with cells transfected with vector alone. To identify genes altered by HPV, fold-change analysis was applied. (A) Dendrogram cluster of YD8 cells, showing two clusters according to the expression profiles of the 1,079 classifier genes selected by applying expression change cut-off of 2-fold. Dendrogram cluster of YD10B cells, showing two clusters according to the expression profiles of the 2,414 classifier genes. (B) Functional classification of differentially expressed genes using categories defined as part of the KEGG pathway database. Pathway list of genes that are differentially expressed in cells transfected with HPV16 E6E7 and cells transfected with vector alone (P<0.05).

We found a significant difference in gene expressions between HPV16 E6E7 transfected cells and cells transfected with vector alone. A total of 1,079 genes were differentially expressed between YD8-V and YD8-E6E7, with 2,414 genes differentially expressed between YD10B-V and YD10B-E6E7 (Fig. 4A). We next sought to identify the molecular mechanisms responsible for these differences in expression by using the pathway mining tool of the Kyoto Encyclopedia of Genes and the Genomes (KEGG) pathway database (http://www.genome.jp/kegg/). This tool maps genes to known pathways and provides a summary of the biological processes affected. Based on this database analysis, we identified 10 pathways that containing ≥10 genetic elements mapped in pathways from 1,079 genes of the molecular signature in YD8 cells and from 2,414 genes of the molecular signature in YD10B cells (Fig. 4B).

As shown in Fig. 4B, the major signaling pathways affected in HPV16 E6E7 transformed cells were identified as focal adhesion, the cytokine-cytokine receptor interaction MAPK signaling pathway, extracellular matrix (ECM)-receptor interaction, the JAK-STAT signaling pathway, the cell cycle and the p53 signaling pathway. The majority of genes involved in focal adhesion, the cytokine-cytokine receptor interaction MAPK signaling pathway and the ECM-receptor interaction, were downregulated in HPV16 E6E7 transfected cells compared with cells transfected with vector alone.

Most of these genes were downregulated in HPV16 E6E7 transfected cells compared with vector alone cells. However, *RAC1, VAV3, GSK3B, THBS3, ITGB4, LAMA3, STAT1, IFI44L, FITM1, IFIH1, SOCS2* and *CDC25B* were expressed at higher levels in HPV16 E6E7 transfected cells compared with vector-alone cells (Table II).

### Apoptosis, cell growth and cell cycle-related gene expression in HPV E6E7 transfected cells

To gain further insight into the molecular mechanisms responsible for differential expression of the markers identified in HPV16 E6E7 transfected cells after their comparison with cells transfected with vector alone, we used qRT-PCR array technology to examine the pattern of expression of 84 genes associated with p53-mediated signal transduction. The array includes p53-related genes involved in the processes of apoptosis, cell cycle progression, cell growth, cell proliferation and cell differentiation and DNA repair. We found significant differences in gene expression between YD8-vector and YD8-E6E7 cells. Four genes were upregulated (i.e., *STAT1, TP73, WT1* and *BCL2*), but seven genes were downregulated (i.e., *ESR1, PRKCA, IGF-1R, EGR1, MSH2, CDKN1A* and *JUN*). The expression of STAT1 was upregulated by 6.47-fold (P<0.05). IGF-1R was downregulated 2.40-fold in YD8-vector compared to YD8-E6E7 (P<0.01) (Table III).

### STAT1 and IGF-1R expression in YD8 cells

**Figure 5:**
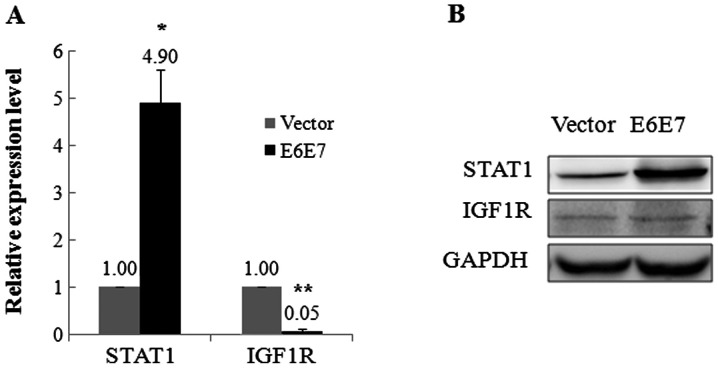
STAT1 and IGF-1R expression in YD8 cells. Validation of the microarray and qRT-PCR array expression in HPV16 E6E7 transfected YD8 cells compared to vector-alone cells using quantitative real-time RT-PCR. (A) The level of *STAT1* and *IGF-1R* mRNAs in YD8-V compared with the levels in YD8-E6/E7. Statistically significant differences between the control and treatment groups are presented as ^*^P<0.05, ^**^P<0.01. (B) The levels of STAT1 and IGF-1R proteins in YD8-V compared with YD8-E6E7. The abundance of GAPDH was determined as a control. The values represent the mean ± SD of each group.

We analyzed similarities in differential gene expression revealed in data from cDNA microarray and qRT-PCR array experiments that compared HPV16 E6E7 transformed cells and cells transfected with vector alone. This analysis revealed that STAT1 and IGF-1R displayed the most significantly differential expression when gene expression was compared in YD8-E6E7 and YD8-V. In order to validate gene expression data obtained using the microarray and qRT-PCR array technologies, we compared the data generated using qRT-PCR and western blot analyses for two genes that are differentially expressed in cells transfected with HPV16 E6E7 or vector alone. As shown in Fig. 5, transcription of *STAT1* was expressed at a higher level in YD8-E6E7 cells compared with YD8-V cells. In contrast, levels of *IGF-1R* transcripts were less abundant in YD8-E6E7 cells compared with YD8-V cells (Fig. 5A). The level of STAT1 protein was also higher in YD8-E6E7 cells than in YD8-V cells. Nonetheless, level of IGF-1R protein was not differentially expressed in YD8-E6E7 and YD8-V cells (Fig. 5B).

### STAT1 and IGF-1R expression in oropharyngeal tumors

Representative examples for the immunohistochemical staining of tumors with low, intermediate and high STAT1 activation are shown in Table IV. STAT1 expression was assessed by immunohistochemistry as described in Materials and methods through evaluation of the percentage of cells with nuclear STAT1 and cytoplasmic STAT1 in HPV-positive/negative cancer. As a result, high-level expression of nuclear STAT1 was slightly higher in HPV-positive than HPV-negative tumors (84 and 88%, respectively) (P=0.18). However, the high-level expression of cytoplasmic STAT1 was significantly lower in HPV-positive tumors than in HPV-negative tumors (27 and 19%, respectively) (P=0.01).

IGF-1R expression was evaluated by determining the percentage of cells with cytoplasmic IGF-1R in HPV-positive/negative cancer. The high-level expression of cytoplasmic IGF-1R was expressed at a low level in HPV-positive tumors compared with HPV-negative tumors (46 and 64%, respectively) (P=0.03) (Table V).

## Discussion

Patients with HPV-positive oropharyngeal cancer show better tumor response to radiation or chemotherapy than patients with HPV-negative cancer (16–18). However, HPV oncoprotein E6 binds and degrades a typically wild-type p53 protein product (24,25). HPV16 infection and p53 mutation may infrequently coexist in a subset of HNSCC, but there is an inverse correlation between HPV16 and disruptive p53 mutation (26). Even if this information has mostly been based upon clinical studies, little is known about the molecular genetics and tumor biology of HPV-positive oropharyngeal cancer characterized as two different subtypes of TP53 mutations.

To address this deficiency, we investigated the biological and molecular changes in two HPV16-negative tongue cancer cell lines: YD8 cells bearing non-disruptive p53 mutation and YD10B cells bearing disruptive p53 mutation (26,27), which had been transfected with the HPV16 E6E7 oncogene. We confirmed the existence of E6E7 DNA amplifications in these stably transfected YD8 and YD10B cells (Fig. 1). Then, we evaluated the expression of the protein products of the tumor suppressor genes, such as p-p53, p-Rb and cell cycle-related genes before and after E6E7 transformation in YD8-E6E7 cells. We observed that most of the proteins were less abundant in YD8-E6E7 cells than in YD8-V cells (Fig. 2). Several studies have suggested that downregulation of p53, pRb and cyclin D1 and upregulation of p16^INK4A^ in HPV-positive head and neck cancer patients are the consequence of functional inactivation of two key tumor suppressor proteins, p-p53 and p-Rb, by the HPV E6 and E7 oncoproteins (18,23,28).

Increased cell proliferative activity in cells transformed with the E6E7 gene may be attributed to the degradation of pRb and p53. E6-mediated degradation of p53 results in the abrogation of the G2/M cell cycle checkpoint upon DNA damage (29). However, in our data, the significantly increased cell population in the G2/M phase in YD8-E6E7 cells may be attributed to p53-mediated growth arrest (Fig. 3A and Table I).

A large body of evidence demonstrated differences in the expression of DNA replication, DNA repair and cell cycle-related genes between HPV-positive and HPV-negative head and neck cancer patients (30–32). Our gene expression profiling study revealed significant changes in the expression of genes not only related to the cell cycle but also to focal adhesion, cytokine-cytokine receptor interaction, MAPK signaling and JAK-STAT signaling (Table II). In addition, a previous study reported that gene expression changes associated with cytokines, growth factors and JAK-STAT signaling pathways as part of an *in vitro* study, which involved HaCaT cells (immortalized human keratinocytes) that had been transfected with the HPV16 genome (33).

Given that the levels of the mRNA and protein products of the cell cycle-related genes, including *p53*, expressed by YD8-E6E7 cells were shown to be altered by cDNA microarray and western blot analyses, we focused on the mRNA expressions of p53 signal pathway-related genes in non-disruptive p53 mutant YD8-E6E7 cells. RT-PCR array analysis revealed that HPV significantly increased the levels of *STAT1, TP73, WT1* and *BCL-2* transcripts and significantly decreased the levels of *IGF-1R, CDKN1A, FGR1, ESR1* and *JUN* (Table III).

Analysis of the results from both cDNA microarray and qRT-PCR array experiments enabled us to select the *STAT1* and *IGF-1R* genes for further analysis. We also verified the higher expression of *STAT1* and the lower expression of *IGF-1R* at the mRNA in the E6E7 transformed cells than in the cells transfected with the vector alone, but the level of IGF-1R protein was not differentially expressed in those cells.

The JAK-STAT pathway is known to be activated in many solid tumors, HNSCC, non-small cell lung cancer (NSCLC) and small cell lung cancer (SCLC) (34). Dysregulation of the JAK-STAT pathway is implicated in tumor formation and progression (35). A recent study has reported that STAT1 expression was decreased in human foreskin keratinocytes (HFK) transfected with wild-type HPV16/31 genomes and E6/E7 (36). In contrast, our data showed that the level of *STAT1* mRNA was higher in YD8-E6E7 cells than in YD8-V cells. We concluded that these conflicting results of STAT1 expression may have originated from the differences between the two target cell lines.

Most of all, previous experiments have been conducted using normal keratinocytes transfected with the HPV viral genome or E6 and E7 oncogenes, these transformed keratinocytes were substantially influence the expression of numerous intracellular target genes, via degradation of tumor suppressor genes such as p53 and pRb (37,38). However, we observed slight decreases in the levels of mRNA and protein that encode p53 when p53-mutated YD8 cells were transformed with the E6E7 gene. Our results were quite different from the previous results showing a significant decrease in the wild-type p53 protein by E6 (24). These data suggest that there is less possibility that E6/E7 oncoproteins control intracellular target genes, including *STAT1*, through the inhibition of p53 expression in the case of HPV-positive oropharyngeal cancer cells bearing non-disruptive p53 mutation.

Analysis of mRNA expression profiling by cDNA micro-array revealed significant changes in the Toll-like receptor genes and genes related to the JAK-STAT signaling pathway-related genes, especially a 2–4-fold upregulation of interferon regulatory factor 7 (IRF7) and interferon-induced genes in the E6E7 transformed cells than in the cells transfected with the vector alone. Considering that a remarkable increase in *STAT1* mRNA was commonly identified in both cDNA microarray and qRT-PCR array when YD8-E6E7 cells were compared with YD8-V cells, we can postulate that the interferon response element in the promoter of the transfected E6 gene binds the interferon regulatory factor, which activates the interferon signal that subsequently accelerates interferon production. As part of a positive feedback loop, extracellular interferon binds to the INF-α receptor and then ultimately activates the JAK-STAT signaling pathway.

Immunohistochemical analysis showed that the expression of STAT1 protein was slightly higher in HPV-positive than in HPV-negative oropharyngeal cases (P=0.18); however, cytoplasmic STAT1 was significantly lower in HPV-positive cases (P=0.03) (Table IV).

This result suggests that STAT1 may be translocated more from the cytoplasm to the nucleus in HPV-positive than in HPV-negative oropharyngeal cancers.

STAT1, STAT3 and STAT5 proteins are frequently overexpressed in head and neck cancer cell lines (39). While STAT1 increases the rates of apoptosis, improves functioning of the immune system and functions as a tumor suppressor by reducing cancer proliferation (40,41), STAT3 maintains the malignant transformation by increasing the proliferation of tumors (39,42).

Levels of *IGF-1R* mRNA were lower in YD8-E6E7 cells than in YD8-V cells and their migration and invasion were also significantly decreased relative to YD8-V cells (Fig. 3B). In addition, immunohistochemical staining of IGF-1R revealed that its abundance in the cytoplasm was remarkably lower in HPV-positive tumors than in HPV-negative tumors when compared with oropharyngeal carcinomas with a high level of IGF-1R expression (Table V).

The IGF-1R protein has been implicated in controlling cellular adhesion, cytoskeletal organization and migration of various solid tumors, including HNSCC, via two major signal pathways: the PI3-K/AKT and RAS/RAF/MAPK pathways (43,44). The early oncoproteins of HPV-16 (E5, E6 and E7) enhance trophoblastic growth by impairing cell adhesion, leading to increased cellular motility and invasive properties (45) and HPV16 E6 increasing the ability of human keratinocytes to adhere on poly(HEME) (46). Our data suggest that decreased invasion activity might be caused by downregulated IGF-1R mRNA.

During the early stage of HPV infection into oropharyngeal mucosal cells, STAT1 expression is suppressed and viral replication is activated with evasion of the immune surveillance (36,47). However, infection of non-disruptive p53-mutated oropharyngeal cancer cells with HPV activates interferon signaling associated with the immune response, which increases rates of STAT1 phosphorylation and apoptosis while reducing the rates of cell proliferation.

In conclusion, we propose that the molecular changes of INF-related and JAK-STAT signals that are triggered by HPV infection might account substantially for the increased sensitivity to chemotherapy or radiotherapy that improves the outcome in HPV-positive oropharyngeal carcinoma cases. Although we did not clearly identify the downstream signals and role of STAT1, our data suggest that activated STAT1 and interferon signals by HPV16 E6 and E7 may play a major role in the relatively favorable prognosis for patients with a non-disruptive p53 mutation, HPV-positive oropharyngeal squamous cell carcinomas. Therefore, upregulated INF-related and JAK-STAT signals likely play a pivotal role in mediating the immune surveillance of HPV-related oropharyngeal cancers and strategies designed to upregulate the immune response hold promise for further improving patient outcomes.

## Figures and Tables

**Table I:** Cell cycle distributions between HPV16 E6E7 transfected cells and vector alone cells.

Cells	Cell cycle distributions			
	G0/G1 (%)	S (%)	G2/M (%)	Sub-G1 (%)
YD8				
E6E7	66.1±1.5^a^	6.3±0.6	18.3±1.2^a^	10.2±1.5
Vector	71.9±1.2	7.6±1.4	13.9±1.4	6.8±0.6
YD10B				
E6E7	79.4±2.8	5.6±1.8	12.3±0.7^a^	1.8±0.4
Vector	74.5±2.8	4.5±1.2	16.4±0.4	3.4±0.6

a P<0.01.

**Table II:** The classification of differentially expressed genes according to signaling pathways in HPV16 E6E7 transfected cells compared with vector alone cells.

KEGG pathway	Regulation	
	Upregulation	Downregulation
Focal adhesion	RAC1, VAV3, GSK3B, THBS3, ITGB4, LAMA3	ITGA11, LAMB1, ITGA2, IGF1R, THBS2, PDGFC, CRKL, COL1A1, TNC, CAV2, BIRC2, PRKCA, COL3A1, COL6A2, COL1A2, PDGFRB, MAPK1
Cytokine-cytokine receptor interaction	CCL26, CXCL16, TNFSF9, CD70, TNFSF10, KITLG, IL28RA	IL4R, IL8, TNFRSF11B, PDGFC, IL6, IL1RAP, IL7R, TNFRSF19, IL1B, CXCL1, IL11, TGFB2, TNFRSF9, PDGFRB
MAPK signaling pathway	RAC1, MKNK2, IKBKG, CD14, BDNF, HSPA2, CDC25B	CRKL, NGF, PPM1B, PRKCA, IL1B, PPP3CB, EVI1, TGFB2, PDGFRB, MAPK1
ECM-receptor interaction	THBS3, ITGB4, LAMA3	ITGA11, LAMB1, ITGA2, THBS2, COL1A1, TNC, COL3A1, COL6A2, COL1A2, CD47
Regulation of actin cytoskeleton	RAC1, VAV3, BAIAP2, CD14, ITGB4	ITGA11, ITGB2, ITGA2, PDGFC, CRKL, DIAPH3, PDGFRB, MAPK1
Jak-STAT signaling pathway	STAT1, IRF7, STAT4, SOCS2, IL28RA, IFI30, IFI35, IFI44L, IFIH1, IFIT1, IFIT2, IFIT3, IFITM1, IKBKG	IL4R, JAK2, IL7R, IL6, IL11, SPRY2
Cell cycle	GSK3B, CDC25B	CDKN2C, CHEK1, CDC45L, CDC2, CDKN1B, MAD2L1, TGFB2, CCNE2
p53 signaling pathway		CHEK1, CDC2, SERPINE1, STEAP3, CCNE2

**Table III:** Apoptosis, cell growth and cell cycle-related genes where were differentially expressed in YD8-E6E7 cells.

Gene symbol	Fold change	P-value
Overexpression		
STAT1	**6.47**	**0.046^a^**
TP73	2.72	0.416
WT1	2.22	0.621
BCL2	2.03	0.450
Underexpression		
ESR1	−3.98	0.454
PRKCA	−2.71	0.129
IGF-1R	**−2.40**	**0.009^b^**
EGR1	−2.35	0.337
MSH2	−2.28	0.349
CDKN1A	−2.10	0.091
JUN	−2.10	0.383

a P<0.05;

b P<0.01.

**Table IV:** Immunohistochemical staining for STAT1.

	HPV-negative		HPV-positive	
	
	Nucleus	Cytoplasm	Nucleus	Cytoplasm
STAT1				
Negative	0 (0)	17 (15)	0 (0)	5 (19)
Low	2 (2)	26 (23)	0 (0)	4 (15)
Intermediate	16 (14)	40 (35)	3 (12)	12 (47)
High	95 (84)	30 (27)	23 (88)	5 (19)
No. of patients (%)	113 (100)	113 (100)	26 (100)	26 (100)

**Table V:** Immunohistochemical staining for IGF-1R.

	HPV-negative	HPV-positive
IGF-1R		
Negative	11 (10)	1 (4)
Low	9 (8)	4 (15)
Intermediate	11 (19)	9 (35)
High	72 (64)	12 (46)
No. of patients (%)	113 (100)	26 (100)
